# Measuring pain in oncology outpatients: Numeric Rating Scale versus acceptable/non acceptable pain. A prospective single center study

**DOI:** 10.1111/papr.13053

**Published:** 2021-07-17

**Authors:** Aniek Anna Julia Martine Willems, Aliaksandr Fedorovich Kudrashou, Maurice Theunissen, Ann Hoeben, Marieke Henrica Johanna Van den Beuken – Van Everdingen

**Affiliations:** ^1^ Department of Medical Oncology, School for Oncology and Developmental Biology (GROW Maastricht University Medical Centre+ Maastricht The Netherlands; ^2^ Department of Anaesthesiology and Pain Management Maastricht University Medical Center+ (MUMC+ Maastricht The Netherlands; ^3^ Centre of Expertise for Palliative Care Maastricht University Medical Center+ (MUMC+ Maastricht The Netherlands

**Keywords:** cancer, evaluation scales, (non) acceptable pain evaluation, Numeric Rating Scale (NRS), oncology outpatients, pain management

## Abstract

**Objectives:**

During all stages of oncologic diseases, pain is still a major problem. The Numeric Rating Scale (NRS) is one of the most frequently used tools for pain assessment, although interpretation is difficult. The main objective of this study is to compare two types of pain evaluation scales: NRS versus (non) acceptable pain evaluation scale. The secondary aim is to analyze a 10% sample of patients indicating non acceptable pain more in‐depth.

**Methods:**

To assess the pain evaluation scales, a prospective observational study, with a nested retrospective in‐depth exploration, was conducted. One‐year data of patients visiting the outpatient clinic of the oncology center of a university hospital were used. Besides the pain scores of all patients, a 10% sample of patients indicating non acceptable pain was analyzed more in‐depth.

**Results:**

During 1 year, a total of 37,580 patients registered at the outpatient clinic, of whom approximately 10% indicated non acceptable pain. The mean NRS of patients indicating non acceptable pain was 6.5 (*n* = 2153). For patients indicating acceptable pain, the mean NRS was 1.6 (*n* = 21,010). Although the presence of pain recorded in the patient record increased substantially over the year, the percentage of reported interventions only slightly increased.

**Conclusion:**

The (non) acceptable pain evaluation seems a valuable addition to the NRS for assessing pain among patients with cancer. As interpretation of the NRS appears to be difficult, using the (non) acceptable pain evaluation is recommended. Moreover, creating awareness among specialists to discuss pain has a positive effect on the amount of pain discussed during consultation.

## INTRODUCTION

During all stages of oncologic diseases, pain is still a major problem. Pain has a severe impact on patients’ quality of life, and numerous psychosocial responses are associated with pain. Over one‐third of patients describe pain related to cancer as distressing or even as an intolerable aspect of their cancer.^1^ An adequate management of pain improves patient‐perceived value of cancer treatment and improves patients’ quality of life and survival.^2^, ^3^ The Numeric Rating Scale (NRS) is one of the most frequently used tools for pain assessment. This unidimensional, validated tool is easily applicable in daily practice and not time‐consuming.^4^ Although the translation from the NRS to mild, moderate, or severe pain according to the criteria formulated by Serlin is generally accepted, the interpretation of the NRS on an individual level remains problematic.^5^ Discrepancies in interpretation of NRS by patients occur because of the individual pain boundaries, expectancies of the treatment, and non‐pain related social and psychological dimensions contributing to the experience of pain.^6^, ^7^ As interpretation of the NRS for patients is difficult, other pain evaluation models need to be considered.^8^ Recent literature^9^, ^10^, ^11^ suggests that an individualized approach using Personal Pain Goals, such as the patients’ acceptable/non acceptable pain, and other multidimensional pain assessment tools, results in better patient satisfaction with regard to pain management. This is attributed to the involvement of patients in the decision making process of their own treatment.

The main objective of this study was to compare 2 types of pain evaluation scales: NRS versus (non) acceptable pain evaluation scale. Furthermore, to assess clues for improvement of care, pain treatment is analyzed in sample groups of oncologic outpatients who reported non acceptable pain.

## MATERIALS AND METHODS

As of 2018, the Healthcare and Youth Inspectorate in the Netherlands stated that “pain” has to be an obligatory measurement in order to meet the quality standards for hospitals.^12^ To meet this obligatory quality measurement, the oncology center of Maastricht University Medical Centre+ (MUMC+) added an electronic questionnaire to the electronic registration procedure. When entering the outpatient clinic, patients first register at the registration column. On this registration column, patients register electronically and also answer 2 questions (in Dutch): (1) What was your pain score last week? And (2) Was this pain acceptable, yes or no? To answer the first question, patients score their mean pain level experienced over the last week, according to the NRS (0–10). If patients need help with the electronic registration process, a volunteer is present for assistance. The scores of the 2 questions save automatically in the medical file of the registered patient. As a result, once the treating specialist opens the medical record, the indicated pain appears immediately.

### Study design

In order to assess the pain evaluation scales, a prospective observational study, with a nested retrospective in‐depth exploration, was conducted. Data of patients visiting the outpatient clinic of the oncology center of MUMC+ in the period from July 11, 2018, until July 11, 2019, were used. To assess the 2 pain evaluation scales, recorded patient data of every first visit was used. To assess the in‐depth exploration, data of the summary of the consultation of patients indicating non acceptable pain was used. Consecutive patients with an oncologic diagnose were included in the study, this includes curatively and palliative patients with the age of 18 years and older. In the academic hospital, every type of cancer is treated. To avoid interference between treating physician and patient, an independent researcher collected the patient data and performed the analyses.

The first intended evaluation had a timespan of almost 5 months (4 months and 20 days). In this period, in a large number of patients indicating non acceptable pain, pain was not discussed during the consultations. In addition, the report of subsequent pain interventions was limited. Therefore, researchers decided to extend the study to 1 year with 3 additional evaluation periods, with a timespan of 2 to 3 months each. Awareness about the importance of pain was created through short lectures during oncology committee meetings in which (oncology) specialists were present.

Study procedures were in conformity with institutional guidelines and adhered to the principles of the Declaration of Helsinki. Patients hospitalized at the academic hospital automatically agree to the use of their medical data for this type of research, in order to evaluate the quality of care/ for quality improvement evaluation of quality of care, unless patients explicitly objected to the use of their medical data for research. Published results cannot lead to identification of individuals. The article was written in accordance with the Strengthening the Reporting of Observational Studies in Epidemiology (STROBE) guidelines.^13^

### Analysis

Besides the NRS pain scores of all patients, a randomly chosen 10% sample of patients (every tenth patient) indicating non acceptable pain was analyzed more in detail by assessing the summary of the consultation in the medical record. In order to assess whether the subject pain was mentioned, the clinical notes of respective visits were searched for words as “pain,” “complaints.” and “suffering.” If one of these words was present, pain was considered to be a discussed subject and explored further in detail. If none of the terms were mentioned, pain was not considered to be discussed and exploration stopped. The pain intensity noted by the specialist in the summary of the consultation was compared to the previously recorded NRS at the registration column. If the pain intensity was mentioned and recorded in the summary of the consultation but was not corresponding to the recorded NRS, the researcher used the following rating scale to estimate and adapt the NRS score: “no pain = 0,” “mild pain = 1–4,” “moderate pain = 5–6,” and “severe pain = 7–10.”^5^ For example, if a patient recorded a 9 (severe pain) at the registration column, but during the consultation the patient corrected his or her given pain score because for instance the pain was not cancer related, the researcher adapted the recorded NRS score to the pain intensity mentioned by the specialist in the summary of the consultation.

Next, it was assessed whether the practitioner carried out an intervention to treat the pain indicated by the patient. Additionally, in case of an executed intervention, the type of intervention was recorded. A distinction was made between the following interventions: “pain medication,” “referral to the pain department,” “referral to specialisms other than the pain department,” and “further diagnostics” or “no new intervention carried out.” Moreover, the type of pain medication was assessed, if applicable. Finally, of the 10% sample, sex and age were registered.

Descriptive analyses were performed using numbers and proportions (%) for categorical data and mean, SD and minimum‐maximum (min‐max) for numerical data. The area under the curve (AUC) and the sensitivity and specificity were calculated for non acceptable pain, using the NRS pain scores of the whole study sample (both patients indicating acceptable and non acceptable pain). Data were analyzed using SPSS version 25 (IBM).

## RESULTS

**FIGURE 1:**
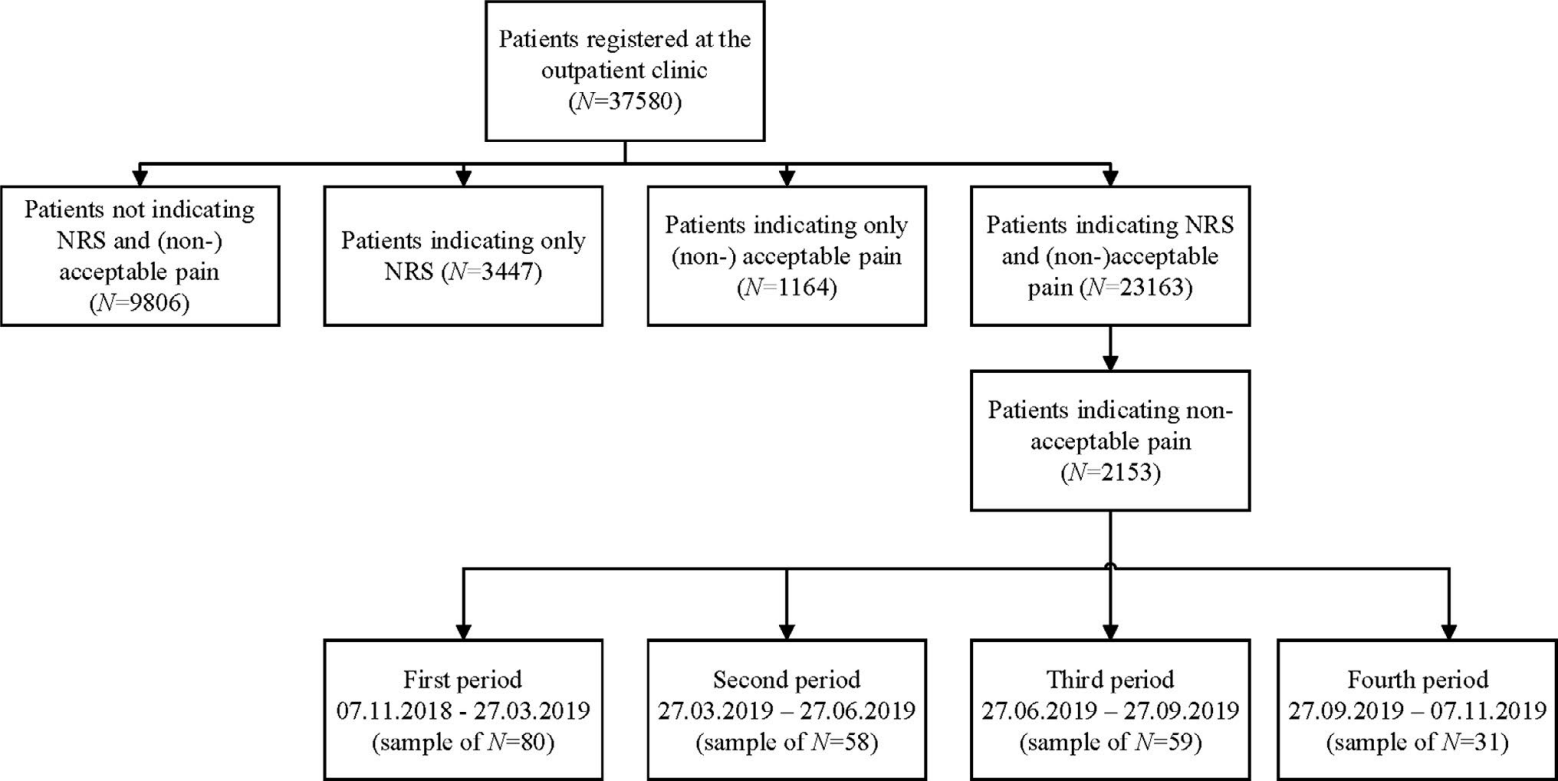
Overall flow diagram of included patients indicating NRS and (non) acceptable pain. Nine percent to 11% of the samples of patients with non acceptable pain were assessed in detail, divided over 4 consecutive periods. NRS, Numeric Rating Scale

**FIGURE 2:**
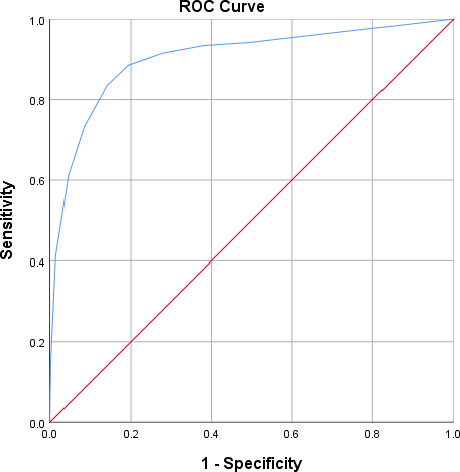
ROC curve for non acceptable pain versus NRS pain score. NRS, Numeric Rating Scale; ROC, receiver operating characteristic

During 1 year, a total of 37,580 patients registered at the outpatient clinic. The overall flow diagram is presented in Figure 1. To assess the cutoff for non acceptable pain on the NRS in this population of oncology patients, the NRS of all patients who scored NRS as well as (non) acceptable of all 4 periods were used (*N* = 23,163). Results show that the mean NRS over the year of patients indicating non acceptable pain was 6.5 (SD 2.4, *n* = 2153). For patients indicating acceptable pain, the mean NRS was 1.6 (SD 2.2, *n* = 21,010). With a cutoff NRS for non acceptable pain set at 3.5 the sensitivity (88.5%) and specificity (80.6%) were most optimal, with an AUC of 0.904, see Figure 2. Of the 2495 patients indicating moderate pain (NRS 5–6), 481 (18.7%) considered this pain as non acceptable. Of the 2273 patients indicating severe pain (NRS 7–10), 1318 (55.5%) considered it as non acceptable.

The secondary outcomes concerning patient characteristics, pain characteristics and interventions of the 10% samples of patients with non acceptable pain are presented in Table 1. Differences in prevalence of non acceptable pain between the different oncology departments were small (data not shown).

**TABLE 1:** Demographics and intervention characteristics, per study period of 10% of patients with unacceptable pain

Non acceptable pain	P1	P2	P3	P4
Sample size	*n* = 80	*n* = 58	*n* = 59	*n* = 31
Pain reported in record	32 (40%)	39 (67%)	49 (83%)	25 (81%)
Intervention executed^a^	21 (68%)	23 (69%)	30 (77%)	16 (72%)
Interventions				
Pain medication	8	11	21	10
Referral pain department	2	1	2	1
Other interventions^b^	11	11	7	5
Sex male	29 (36%)	26 (45%)	19 (32%)	15 (48%)
Age in years	60.5 (28–92)	62.7 (30–85)	63.4 (30–91)	63.5 (27–86)

^a^ Percentage of executed interventions for patients indicating non acceptable pain were pain was reported in the patients’ record.

^b^ Referral to specialisms other than the pain department, further diagnostics, and no new intervention carried out.

For patients indicating non acceptable pain, several interventions were carried out. During the 4 periods, medication was given in 56% of the cases, referral to the pain team in 7% of the cases, and in 38% of the cases treatment was categorized as “other interventions.” The numbers and percentages of reported interventions for each period are presented in Table 1.

The proportion of patients with non acceptable pain with whom pain was discussed during a consultation increased from 40% in the first period, to 81% in the fourth period. Although the presence of pain recorded in the patient record increased substantially, the percentage of reported interventions slightly increased from 68% to 72% of all the patients indicating non acceptable pain.

## DISCUSSION

This study shows that using the (non) acceptable pain evaluation scale seems a valuable addition to the NRS for assessing pain among patients with cancer. According to current literature, using an individualized approach, such as the (non) acceptable pain evaluation results in better patient satisfaction with regard to pain management.^9^, ^10^, ^11^ Because interpretation of the NRS can be difficult for patients as well as care givers,^5^ using the (non) acceptable pain evaluation is recommended. Despite increased attention to cancer pain, pain prevalence in patients with cancer has not decreased over the last decade. One third of patients with cancer on anticancer therapy and half of patients with advanced disease not on anticancer therapy still suffer from moderate to severe pain according to the NRS (scores between 5 and 10). In the patient cohort presented in this study, ~ 10% of the patients are suffering from non acceptable pain. Cancer‐related pain is therefore considered as undertreated.^14^ This study shows that creating awareness among specialists to discuss pain during a consultation has a positive effect on the amount of pain discussed during consultation. Moreover, teaching specialists how to discuss pain during a consultation results in less undertreated pain among patients with cancer.^15^ However, despite the increased awareness among specialists to discuss patients’ perceived pain, the amount of executed interventions only increased slightly. Common barriers for specialists to assess and manage pain include lack of knowledge and skill, and the reluctance of specialists to prescribe opioids.^15^

With a cutoff point for non acceptable pain set at 3.5, the sensitivity (88.5%) and specificity (80.6%) was most optimal for cancer‐related pain, with an AUC of 0.904. However, this would imply that still 11.5% of the patient population with non acceptable pain would be unrecognized. Moreover, the mean NRS of patients indicating acceptable pain lies between 3.5 and 6.5 and is thus overestimated. Using a unidimensional pain assessment is therefore not recommended. When a unidimensional pain assessment scale needs to be used for practical reasons anyhow, the (non) acceptable pain evaluation scale is of preference.

### Strengths and limitations of the study

Based on a large study population insight was obtained in the prevalence of non acceptable pain in oncology outpatients. In addition, it was possible to assess differences between perceived pain using NRS versus (non) acceptable evaluation scale, including the establishment of an optimal cutoff for the NRS. By prospectively measuring 4 periods, trends over the year were made visible. However, patients with chronic pain could not be excluded. In addition, patients who visited the outpatient clinic more than one time a year were not excluded. As a result, in the calculation of the AUC, some patients are taken into account multiple times. Finally, whereas the pain scores were assessed prospectively, data on pain treatment were retracted retrospectively from the medical files. Therefore, information bias cannot be excluded.

### Implications for practice

The results confirm that the evaluation of (non) acceptable pain is a valuable addition to the NRS for assessing patients’ perceived pain. In addition, by creating awareness among specialists about pain by providing periodic feedback, the amount of consultations in which pain is discussed and treated can be increased significantly.

### Suggestions for further research

As pain is still a major underdiscussed topic during oncology consultations, not all relevant pain characteristics could be extracted from the summary of the consultation in the medical file. On top of that, within 30% of the patients suffering from non acceptable pain, no intervention was carried out. To gain more in‐depth information about the rationale for not carrying out an intervention, further research within the 30% of the patients perceiving non acceptable pain is necessary. In order to conduct this in‐depth research about pain among patients with cancer, specialists need to secure more detailed information concerning pain characteristics and treatment, such as the type of pain, if the perceived pain is tumor‐ or treatment‐related, if is pain considered transient, if patients are in their curative or palliative state, and who executed the intervention.

## CONCLUSION

The (non) acceptable pain evaluation seems a valuable addition to the NRS for assessing pain among patients with cancer. As literature shows that the personalized “(non) acceptable pain evaluation” results in better patient satisfaction with regard to pain management, and interpretation of the NRS appears to be difficult, using the (non) acceptable pain evaluation is recommended. Moreover, creating awareness among specialists to discuss pain has a positive effect on the amount of pain discussed during consultation.

## CONFLICT OF INTEREST

The authors declare no conflict of interest.
